# Workplace ostracism and employee wellbeing: A conservation of resource perspective

**DOI:** 10.3389/fpubh.2022.1075682

**Published:** 2023-01-12

**Authors:** Long-mei Wang, Lei Lu, Wei-lin Wu, Zi-wei Luo

**Affiliations:** ^1^Zhongshan Polytechnic, Zhongshan, China; ^2^School of Business, Macau University of Science and Technology, Taipa, Macao SAR, China

**Keywords:** workplace ostracism, forgiveness climate, emotional exhaustion, employee wellbeing, conservation of resource theory (COR)

## Abstract

**Introduction:**

As a common phenomenon of workplace ostracism in corporate management, it is urgent to clarify how it affects employee well-being.

**Methods:**

Based on Conservation of Resource Theory, this study investigates the mechanisms of workplace ostracism on employee well-being and examines the mediating role of emotional exhaustion and the moderating role of team forgiveness climate by surveying 282 employees from 68 companies in mainland China.

**Results:**

The results show that (1) workplace ostracism negatively affects employee well-being; (2) emotional exhaustion plays a mediating role between workplace ostracism and employee well-being; (3) team forgiveness climate weakens the negative effect of workplace ostracism on emotional exhaustion and negatively moderates the indirect effect of workplace ostracism on employee well-being through emotional exhaustion.

**Discussion:**

It tries to provide theoretical basis and practical guidance for eliminating the negative effects of workplace ostracism and focusing on employee well-being.

## 1. Introduction

Workplace ostracism is prevalent in management practice. In the United States, a survey of 262 workplace employees revealed that nearly 70% of employees felt they had been ostracized by their leaders or co-workers at work (1). Similarly, in China, a month-long survey of more than 10,000 employees conducted by Zhaopin Ltd., a workplace research company, revealed that nearly half of employees believe they are ostracized at work (2). The phenomenon that individuals perceive being neglected, excluded, or rejected by others in the workplace is becoming an increasingly common and serious problem, with damaging consequences for victims (3). In the Chinese context, workplace ostacism had a negative impact on victims' physical and psychological health (4), work attitude (3), workplace behavior (5), work performance (6), and innovation (7), and the impact intensity decreases successively (8). As China's economy takes off and material living standards improve, people are becoming more and more conscious of their physical and psychological health and wellbeing experiences. The term “wellbeing” has gradually appeared in organization management (9) as a relatively broad concept that includes three aspects: work, psychology, and life (10). For employees, wellbeing is closely related to individual work engagement (11), life satisfaction (12), and occupational health (13). For enterprises, employees with a sense of wellbeing are more likely to achieve high levels of work performance, which is conducive to improving enterprise efficiency (14). In China, where the “people-oriented” management mode is advocated, paying attention to employees' physical and psychological health and improving their wellbeing are conducive to a win–win situation for both employees and the company (15, 16). However, workplace ostracism, as a widespread and relatively new phenomenon, is a difficult issue for organizations to solve, whether it damages employee wellbeing or how it affects their wellbeing.

Ostracism, the act of being neglected or excluded, significantly influences behavior toward others (17). The workplace is a social setting where ostracism occurs at a high frequency. Workplace ostracism is the degree to which employees feel ignored or excluded by other members of the workplace (3). Workplace ostracism will significantly increase employees' psychological stress, lead to health problems, and even trigger workplace deviant behaviors that can impair the normal operation of the organization (18). Given the prevalence of workplace ostracism and its serious harmful effects on employees and organizations, and the influence of Chinese “people-oriented” management mode, the focus is on the physical and psychological health of employees. This study aims to investigate the mechanism of workplace ostracism on employee wellbeing.

Workplace ostracism, as a stressful situation for employees, can be understood to some extent as a threat of resource loss. However, the conservation of resource theory (COR) initially emerged as a stress theory, emphasizing the cultivation, retention, and maintenance of resources (19). The conservation of resource theory suggests that individuals have the incentive to retain existing resources and acquire new ones and also take the initiative to create resource surpluses to offset the potential loss of resources in the future (19). As a potential threat of resource loss, workplace ostracism tends to trigger tension and stress in individuals (19, 20). At the same time, workplace ostracism, as a stressful situation that generates the threat of resource loss and resource loss, brings greater physical and psychological stress to the ostracized person, leading to tension and anxiety, generating emotional exhaustion, which in turn destroys their good experiences in work and life and affects employee wellbeing.

Although workplace ostracism will trigger negative emotions and experiences, its effects will also be influenced by situational factors. Employees' emotions, psychological states, and workplace behaviors are interfered with by the team climate (21, 22). Team forgiveness climate as an organizational phenomenon is manifested by team members' empathy and benevolence for the faults or failures of others (23). It can be interpreted as a shared perception of team support, encouragement, and expectations in the face of conflict or failure (24). The care and understanding given to employees by the team forgiveness climate, as a gaining resource, help to alleviate and compensate for the loss of resources caused by employees being excluded or isolated.

Therefore, in order to make up for the research gap by focusing on employee wellbeing from negative perspectives, based on the aforementioned analysis, this study investigates the impact of workplace ostracism on employee wellbeing in the context of Chinese organizational workplaces by using the conservation of resource theory as the basis for a survey of teams and employees in mainland Chinese companies. At the same time, employees' emotional exhaustion is considered a resource variable of individual traits, and the mediating role of emotional exhaustion between workplace ostracism and employees' wellbeing is discussed. It attempts to reveal the specific mechanism of how workplace ostracism affects employee wellbeing from the perspective of psychological traits. Moreover, considering that team climate has an intervening effect on employees' psychology and behavior in the organization, team forgiveness climate is used as a contextual variable in this study to discuss the boundary conditions of workplace ostracism on employee wellbeing, to expand the understanding of the impact of workplace ostracism and its mechanism of action, and to provide inspiration and reference to the corresponding management practices.

## 2. Theory and hypothesis

### 2.1. Workplace ostracism and employee wellbeing

Employee wellbeing refers to employees' evaluation of the overall quality of their career and work experience (25). Zheng et al. divide wellbeing into three dimensions (10): work, psychological, and life wellbeing, which more comprehensively reflect the wellbeing status of individuals.

As a common social phenomenon, workplace ostracism behaviors include silent treatment, neglection, and avoidance of contact (18). Workplace ostracism is a specific form of ostracism, a phenomenon in which individuals perceive that they are neglected or rejected by others in the workplace, a kind of emotional office abuse (3, 26). When employees suffer from ostracism, it will cause them to suffer from self-esteem, generate negative emotions, reduce work efficiency (27, 28), and hinder the generation of wellbeing (29). First, when employees perceive ostracism or isolation in the team, interpersonal stress will be generated, which will affect employees' engagement and satisfaction with their work and reduce their wellbeing at work. Second, ostracism will cause employees' anger, anxiety, and even negative psychological feelings, such as self-doubt and self-denial, leading to excessive consumption of their psychological resources and ultimately reducing their psychological wellbeing (30). Third, as producers of “emotional office abuse,” ostracized employees are perceived as “outsiders” and have certain prejudices (31). On the one hand, ostracized employees have the feeling of “not fitting in,” which increases the burden of getting along with colleagues. On the other hand, it is difficult for the ostracized employees to obtain care and help from the organization members in their work and life, which reduces their wellbeing in work, psychology, and life and negatively affects employees' sense of happiness. Finally, workplace ostracism is often associated with punitive measures, implying employees have done something wrong, thus, reducing employees' organizational commitment, causing the ostracized individuals to be less engaged in their work (32, 33), undermining employees' good experiences at work, in life, etc., and ultimately inhibiting employee wellbeing (18, 34). At the same time, according to the belongingness theory, workplace ostracism makes it difficult for employees to belong to the work team, which affects employees' psychology, behavior, and work-related effects (18, 35, 36) and also affects employees' sense of wellbeing (37, 38). Therefore, Hypothesis 1 is proposed:

Hypothesis 1: Workplace ostracism negatively affects employee wellbeing.

### 2.2. The mediating role of emotional exhaustion

Emotional exhaustion refers to an individual's loss of interest and enthusiasm for people, things, and objects around, and a feeling of physical and psychological exhaustion and energy depletion, reflecting the individual's negative emotional experience and state, and is often regarded as a resource shortage (39, 40). According to the conservation of resource theory, the loss of resources puts more pressure on the individual and the individual has to invest more resources to offset further resource losses (41). Once a loss of resources has occurred, it may lead to a continuous loss, resulting in a spiral of negative effects (42, 43).

Workplace ostracism will cause the depletion of employees' emotional and psychological resources, which will lead to emotional exhaustion (44, 45). First, according to the conservation of resource theory, individuals have the motivation to protect, maintain, and obtain their own resources and become stressed and exhausted when their resources are reduced or threatened. If an individual's depleted resources are not replenished in a timely manner, it can easily lead to emotional exhaustion (46). Second, when employees suffer from workplace ostracism, they are cut off from their emotional connections with other employees. Individuals need social interactions as a way to communicate emotionally to strengthen their emotional resources and maintain physical and psychological health (47). When the need for shared emotions is not met, emotional resources are lost and, thus, suffer emotional exhaustion (35). Third, ostracism is a major challenge for individuals to hold resources and can reduce the resources held by individuals (48). On the one hand, individuals will consume their psychological resources in the process of handling and coping with ostracism. On the other hand, in their daily work, employees need to contact each other in order to obtain external resources, and it is difficult for individuals who suffer from ostracism to replenish their resources from others, so they can only deplete their own resources. Subsequently, their own resources become less, and they may become stressed, anxious, and exhausted. Finally, being ostracized means that individuals lack reliable interpersonal networks, making it difficult for employees to trust members of the organization and lack a sense of security. In this low trust and insecure workplace environment, individuals who suffer from ostracism will take a more cautious stance to maintain relationships with surrounding employees, which will consume more of their own resources and increase the self-depletion of resources, leading to emotional exhaustion in the long run.

Emotional exhaustion, a typical manifestation of psychological overwork, can further lead to a lack of emotional and psychological resources and reduce employees' sense of wellbeing. On the one hand, according to the affective event theory, negative emotional events will affect individuals' emotional responses, and at the same time, individuals' emotional responses will further influence their attitudes and behaviors (49, 50). On the other hand, emotionally depleted employees are more likely to show dissatisfaction and aversion to their jobs and lives, resulting in turnover intention and lower life satisfaction (45). Emotional exhaustion reflects individual psychological feelings and health status and is a negative indicator of employee wellbeing (51). When employees perceive workplace ostracism, as a negative emotional event, based on the social exchange theory, it triggers a depletion of their emotional resources and ultimately leads to a decrease in wellbeing (26, 36). Therefore, this study infers that the higher the level of workplace ostracism an individual suffers, the higher degree of emotional exhaustion, which in turn will affect the individual's wellbeing and inhibit employee wellbeing. Therefore, Hypothesis 2 is put forward as follows:

Hypothesis 2: Emotional exhaustion mediates the relationship between workplace ostracism and employee wellbeing.

### 2.3. The moderating role of forgiveness climate

Forgiveness is an individual's conscious effort to relieve resentment and abandon potential retaliation in the face of offense or aggression (52). With the intensification of social competition, various conflicts burst out (53). In the workplace, employees have conflicts over job resources and promotion opportunities. At this point, if the organization fails to develop an effective forgiveness climate, employees will inevitably waste resources and time in responding to conflict, failing to engage in their work and affecting the output of work performance. Team forgiveness climate refers to the perception of team support when employees show kindness and altruism in the face of conflict and failure, which is mainly reflected in the team's tolerance of fault or negligent behavior (24).

Team climate serves as an important situational variable when working in the workplace. When employees suffer from ostracism, their psychological resources will be lost. At this time, the team's forgiveness climate can alleviate the loss of psychological resources when employees cope with ostracism. According to the resource conservation theory (54), the forgiveness climate of the team, as a resource supplement, helps to reduce the generation of emotional exhaustion (55). First, after a forgiveness climate is formed in the team, team members are more likely to maintain optimistic and positive emotions (56), instead of complaining or blaming others. At this point, even if the employee suffers from workplace ostracism, it will counteract the negative emotions and thus will not easily cause emotional exhaustion. In addition, the forgiveness climate of the team can improve the interpersonal relationship between team members, making them friendly and trusting, which is conducive to cooperation and mutual assistance among the team members (57). At this time, even if employees suffer from rejection and emotional resources are destroyed, the existence of such a trust-friendly climate will alleviate or offset the generation of negative emotions (58). Finally, in view of the social exchange theory, when a forgiveness climate releases a signal similar to the failure of tolerance, team members show more communication and cooperation in return for the team's tolerance, resulting in more active behaviors and positive expressions (59). Even when employees suffer from ostracism, they will try to minimize the generation of negative emotions in order to repay the team's tolerance so as not to affect their work engagement. Therefore, this study infers that when individuals feel a climate of forgiveness, those who suffer from workplace ostracism will suppress the generation of emotional exhaustion. Thus, Hypothesis 3 is supported:

Hypothesis 3: Forgiveness climate plays a negative moderating role in the relationship between workplace ostracism and emotional exhaustion. That is, the higher the level of forgiveness climate perceived by employees, the weaker the positive effect of workplace ostracism on emotional exhaustion. Conversely, the effect is stronger.

### 2.4. Mediation model with moderation

Integrating Hypotheses 2 and 3, this study proposes a mediated model with moderation. It can alleviate the emotional exhaustion caused by workplace ostracism and restrain the decrease in employee wellbeing, that is, a forgiveness climate will suppress the negative effects of workplace ostracism on employee wellbeing through emotional exhaustion. In view of this, Hypothesis 4 is proposed:

Hypothesis 4: The forgiveness climate negatively moderates the negative effect of workplace ostracism on employee wellbeing through emotional exhaustion. The higher the level of forgiveness climate perceived by employees is, the weaker this indirect effect is. On the contrary, this indirect effect is stronger.

**Figure 1:**
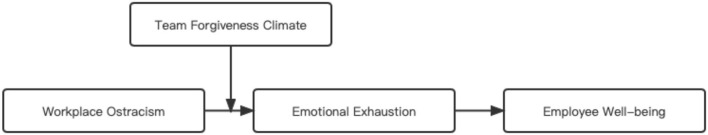
Research framework—a theoretical model.

Thus, emotional exhaustion is introduced as the mediating variable and forgiveness climate as the moderating variable. According to the logical relationship, four hypotheses are put forward to build a theoretical model to study the impact of workplace ostracism on employee wellbeing. The research framework of this article is shown in Figure 1.

## 3. Research methods and tools

### 3.1. Research subjects and collection procedures

Before the survey, the participating employees were communicated and informed that there were no right or wrong answers for all respondents, and the anonymity and confidentiality of the questionnaire were promised. To reduce common method bias, the paired sample of employees and leaders was adopted in this study. Also, to avoid common method bias, the same group of participants was given questionnaires at three time points. Each time interval was 1 month with the entire survey lasting 3 months (June–August 2021). First, the HR department randomly selects the department supervisors who participate in the survey, and the supervisors randomly select 3–6 direct subordinates; then, the supervisors' questionnaires are matched and numbered with the employees' questionnaires. At the first time point, the supervisor questionnaire for forgiveness climate and employee questionnaire 1 for workplace ostracism were distributed; employee questionnaire 2 about employee emotional exhaustion was distributed at the second time point, and employee questionnaire 3 was distributed at the third time point about employee wellbeing.

After the investigation is completed, the last four digits of the phone number of “leader-employee” will be used as the matching basis. In this research, a total of 80 supervisor questionnaires and 350 employee questionnaires were distributed, of which 68 and 282 were collected, respectively, after eliminating invalid and unmatched ones with an effective recovery rate of 80.57%. Employee sample characteristics are as follows: There were 139 male employees (49.3%), 143 female employees (50.7%), 62 unmarried employees (22%), and 220 married employees (78%). The average education level is college, the average age is 38.83 years old, the average working years is 4.97 years, and the average working hours per week is 49.83 h.

### 3.2. Measuring tools

To ensure the reliability and validity of the questionnaire, established scales were used for reference in this study. The English scales were accurately translated into Chinese in terms of the standard translation and back-translation procedure (60) before investigation and research and were repeatedly checked before distribution. A 5-point Likert scale (1–5 in the questionnaire indicates “strongly disagree” to “strongly agree”) was used throughout the study.

#### 3.2.1. Workplace ostracism

A questionnaire was applied to measure the degree of workplace ostracism perceived by employees with 10 items in total according to the scale compiled by Ferris et al. (3). Examples of questions are “Your greetings have gone unanswered at work” and “You noticed others would not look at you at work.” The scale has a Cronbach's alpha coefficient of 0.906 in this study.

#### 3.2.2. Emotional exhaustion

A 3-item scale developed by Boswell et al. (39) was used. Examples of questions are “I feel emotionally drained from my work” and “I feel exhausted when I think about having to face another day on the job.” This scale has a Cronbach's alpha coefficient of 0.762 in this study.

#### 3.2.3. Forgiveness climate

There are four questions with reference to Cox's (52) forgiveness climate scale. Examples of questions are “We can forgive the faults and mistakes of team members” and “we don't hold grudges in a team.” This scale has a Cronbach's alpha coefficient of 0.952 in this study.

#### 3.2.4. Employee wellbeing

The wellbeing scale with 18 items was compiled by Zheng et al. (10), covering life wellbeing, work wellbeing, and psychological wellbeing. Examples of questions are “Most aspects of my life are very close to my ideal,” “I always find ways to enrich my work” and “As years went by, I feel that I have grown a lot.” This scale has a Cronbach's alpha coefficient of 0.958 in this study.

#### 3.2.5. Control variables

Based on previous studies, gender, age, and education have been found to influence employee wellbeing (10). In addition, employees' weekly working hours and length of service also make a difference in their perceptions of wellbeing (61, 62). To more accurately validate the model, gender, age, education, length of service and the weekly working hour was measured as control variables in this study.

### 3.3. Analysis tools

In this study, SPSS 21.0 was employed for Harman's one-way test, descriptive statistics, correlation analysis, and multiple regression analysis, and Amos 21.0 was used for the confirmatory factor analysis. For testing the mediating effects, the three-step method of Baron and Kenny (63) was used and combined with the Bootstrap technique (using the PROCESS program) (64) to estimate confidence intervals for mediating effects. In testing for mediators with moderation, this study tested the significance of the values and differences of indirect effects under high and low moderating variables relied on Edwards and Lambert's (65) study and integrated with the bootstrap technique.

## 4. Research results

### 4.1. Common method deviation test

In this research investigation, the multi-stage fill-in approach, suggested by Podsakoff et al. (66), was followed to control for possible common method bias from the methodological level (66). At the data level, Harman's one-way test of the collected data revealed that the percentage of explained variance by the first factor was 34.38%, which is < 40% criterion (66). In addition, as can be seen in Table 1, the fitting results of the confirmatory factor analysis of the one-factor model also failed the test (χ^2^ = 3,064.72, df=560, RMSEA = 0.13, SRMR = 0.17, CFI = 0.56, and TLI = 0.59). Therefore, there is no serious common method bias among the variables in this study.

**Table 1:** Results of confirmatory factor analysis (*N* = 282).

**Model**	**χ2**	**df**	***Δχ*2**	**RMSEA**	**SRMR**	**CFI**	**TLI**
Four-factor model (hypothesis)	691.58	554		0.03	0.04	0.98	0.98
Three-factor model (A+B)	890.81	557	199.23^***^	0.05	0.07	0.94	0.94
Two-factor model (A+B+D)	1,904.41	559	1,212.83^***^	0.09	0.11	0.77	0.78
One-factor model (A+B+C+D)	3,064.72	560	2,373.14^***^	0.13	0.17	0.56	0.59

A, workplace ostracism; B, emotional exhaustion; C, forgiveness climate; D, employee wellbeing; “+” indicating integration.

### 4.2. Confirmatory factor analysis

In this study, the following fit index was selected to judge the fit of the model, including the chi-square difference must reach a significant level, the root means square error of approximation (RMSEA) must be < 0.08, and the comparative fit index (CFI) and Tucker–Lewis index (TLI) must be > 0.9. A series of competing models were compared in this study, and the results of the analysis are shown in Table 1. As can be seen in Table 1, the model fit of the four-factor model (χ2 = 691.58, df = 554, RMSEA = 0.03, SRMR = 0.04, CFI = 0.98, and TLI = 0.98) was better than other competing models in this study. Moreover, all the fit indicators of the four-factor model passed the test. In the view of above, all the variables in this study were found to be distinguishable.

### 4.3. Correlation analysis

The results of the correlation analysis between control variables and variables are shown in Table 2. As can be seen in Table 2, there is a significant negative relationship between workplace ostracism and employee wellbeing (r =- 0.36, *p* < 0.001), which provides preliminary support for exploring the negative prediction of workplace ostracism on employee wellbeing.

**Table 2:** Mean values, standard deviations, and correlation coefficients of variables (*N* = 282).

**Variables**	**Mean Values**	**Standard deviation**	**1**	**2**	**3**	**4**	**5**	**6**	**7**	**8**
1 Gender	0.51	0.5								
2 Age	38.83	8.15	0.07							
3 Education	14.7	1.99	−0.06	0.70^***^						
4 Length of service	4.97	2.67	0.09	0.41^***^	−0.34^***^					
5Weekly working hour	49.83	6.32	0.11	0.16^**^	−0.09	0.24^***^				
6 Workplace ostracism	2.84	0.95	0.01^*^	0.19^**^	−0.19^**^	0.11	0.02			
7 Emotional exhaustion	2.87	1.06	0.06	−0.13^*^	0.1	−0.09	−0.02	0.27^***^		
8 Forgiveness climate	4.14	0.96	0.04	−0.01	−0.01	−0.07	−0.04	−0.02	0.08	
9 Employee wellbeing	3.29	0.96	−0.04	−0.01	0.08	0.06	0.01	−0.36^***^	−0.31^***^	0.01

^***^p < 0.001, ^**^p < 0.01, and ^*^p < 0.05.

### 4.4. Hypothesis testing results

#### 4.4.1. Test results of the main effect

According to Model 5 in Table 3, workplace ostracism has a significant negative relationship with employee wellbeing (β = −0.37, *p* < 0.001). Hypothesis 1 is supported.

**Table 3:** Hypothesis testing model.

**Variables**	**Emotional exhaustion**			**Employee wellbeing**			

	**Model 1**	**Model 2**	**Model 3**	**Model 4**	**Model 5**	**Model 6**	**Model 7**
**Control variable**						
Gender	0.07	0.07	0.07	−0.04	−0.04	−0.03	−0.03
Age	−0.11	−0.14	−0.13	0.05	0.09	0.06	0.06
Education	0.02	0.06	0.05	0.15	0.1	0.12	0.11
Length of service	−0.04	−0.05	−0.05	0.1	0.11	0.1	0.1
Weekly working hour	0	0.01	−0.01	−0.01	−0.01	−0.01	−0.01
**Independent variable**						
Workplace ostracism		0.32^***^	0.31^***^		−0.37^***^	−0.29^***^	−0.29^***^
**Mediating variable**						
Emotional exhaustion						−0.23^***^	−0.24^***^
**Moderating variable**						
Forgiveness climate			0.1				0.04
**Interaction items**						
Workplace ostracism^*^Forgiveness climate			−0.12^*^				−0.05^*^
R^2^	0.02	0.12	0.14	0.02	0.15	0.19	0.19
F	1.33	29.70^***^	12.19^***^	1.03	40.93^***^	29.16^***^	14.78^***^

^***^p < 0.001, ^**^p < 0.01, and ^*^p < 0.05.

#### 4.4.2. Test results of mediating effect

As shown by Models 2, 6 in Table 3, workplace ostracism presents a significant positive relationship with emotional exhaustion (β = 0.32, *p* < 0.001) and emotional exhaustion showed a significant negative relationship with employee wellbeing (β = −0.23, *p* < 0.001), verifying the indirect effect of workplace ostracism on employee wellbeing through emotional exhaustion. To clarify this indirect effect again, the Bootstrap method test was used in this study (64). The Bootstrap method test for the mediating effect is shown in Table 4, where both the direct and indirect effects of workplace ostracism and employee wellbeing do not include zero at the 95% confidence interval. Therefore, it can be confirmed that emotional exhaustion in the relationship between workplace ostracism and employee wellbeing played a partially mediating role in the relationship between workplace ostracism and employee wellbeing. Hypothesis 2 is supported.

**Table 4:** Bootstrap test for mediating effects.

**Mediating effect**	**Effect value**	**Standard error**	**95% of confidence interval**	
			**Lower confidence limit**	**Upper confidence limit**
Indirect effect	−0.07	0.02	−0.12	−0.03
Direct effect	−0.30	0.06	−0.41	−0.18

Bootstrap sample size N = 5,000.

#### 4.4.3. Test results of moderating effects

**Figure 2:**
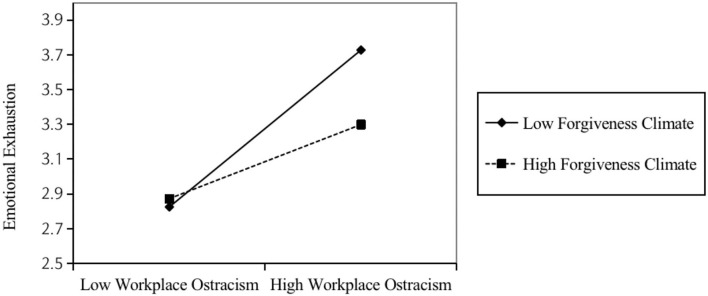
Moderating role of forgiveness climate between workplace ostracism and emotional exhaustion.

Verifying the moderating effect of forgiveness climate. As shown by Model 3 in Table 3, the interaction term between workplace ostracism and forgiveness climate displayed a significant negative relationship with emotional exhaustion (β = −0.12, *p* < 0.05). Also, the Bootstrap method test for the moderating effect is shown in Table 5, at a confidence interval of 95%, where the indirect effect of workplace ostracism on emotional exhaustion is higher in a low degree of forgiveness climate (with effect value of 0.48) and lower in a high degree of forgiveness climate (with effect value of 0.24). To further clarify this moderating effect, the study was determined by using the method provided by Aiken et al. (67) to regulate the high and low levels of the moderating variables. According to Figure 2, the positive relationship between workplace ostracism and emotional exhaustion is weaker at higher levels of the forgiveness climate. Hypothesis 3 is, thus, supported.

**Table 5:** The bootstrap test of the moderating effect with forgiveness climate.

**Moderating effect**	**Effect value**	**Standard error**	**95% of confidence interval**	

			**Lower confidence limit**	**Upper confidence limit**
Low (−1SD)	0.48	0.09	0.30	0.65
Medium	0.35	0.06	0.22	0.48
High (+1SD)	0.24	0.09	0.07	0.41

Bootstrap sample size N = 5,000.

#### 4.4.4. Test results of mediating effects with moderation

This study aims to verify whether a forgiveness climate moderates the indirect effect of workplace ostracism on employee wellbeing through emotional exhaustion. This study also employed the Bootstrap method to examine the effect values of indirect effects at high levels vs. low levels of moderating variables (65). Table 6 illustrates that in a high degree of forgiveness climate, the indirect effect of workplace ostracism on employee wellbeing through emotional exhaustion is −0.05, whose value is [−0.09, −0.01] at a 95% confidence interval. Under the condition of a low level of forgiveness climate, the indirect effect of workplace ostracism on employee wellbeing through emotional exhaustion is −0.10, with the value of [−0.15, −0.05] at a 95% confidence interval. In conclusion, the higher the degree of forgiveness climate, the weaker the indirect effect of workplace ostracism on employee wellbeing through emotional exhaustion. Hypothesis 4 is, thus, supported.

**Table 6:** Bootstrap test for mediating effects with moderation.

**Independent variable**	**Moderating variable (forgiveness climate)**	**Indirect effect**	**Standard error**	**95%of confidence interval**	

				**Lower confidence limit**	**Upper confidence limit**
Workplace ostracism	Low (−1SD)	−0.10	0.03	−0.15	−0.05
	Medium	−0.07	0.02	−0.12	−0.03
	High (+1SD)	−0.05	0.02	−0.09	−0.01

Bootstrap sample size N = 5,000.

## 5. Conclusion

From the perspective of resource conservation, this study uses matched data from supervisors and subordinates, adopts a multi-stage research method, introduces emotional exhaustion as a mediating variable and forgiveness climate as a moderating variable, and deeply analyzes the mechanism of the effect of workplace ostracism on employee wellbeing. This study not only expands the research in the field of workplace ostracism but also clarifies the causes of employee wellbeing.

## 6. Discussion

### 6.1. Theoretical implications

First, this study takes the perspective of resource consumption and uses the conservation of resource theory to explain the effects of workplace ostracism. It confirms that emotional exhaustion plays a role in bridging workplace ostracism and employee wellbeing, provides a new theoretical perspective and interpreting path to explain how workplace ostracism affects employee wellbeing, and expands a new angle to study employee wellbeing (68, 69). Meanwhile, workplace ostracism is seen as a stressor that depletes employees' emotional resources and negatively affects their attitudes and behaviors (8).

Second, this study examines the borderline role of workplace ostracism in affecting employee wellbeing. The forgiveness climate acts as a negative moderator between workplace ostracism and emotional exhaustion. The perception of the team forgiveness climate by employees is regarded as a resource supplement, which can alleviate the negative impact caused by ostracism (70). This conclusion has profound implications for understanding workplace ostracism and emotional exhaustion.

Finally, this study focuses on employee wellbeing. The relationship between the role of workplace ostracism on employee wellbeing is examined in terms of three aspects of employees' work, psychology, and life wellbeing. Also, this study verifies that when employees perceive ostracism, there is a loss of psychological resources that causes emotional exhaustion (48, 71), which reveals the mechanism of how negative workplace behaviors affect employee wellbeing (72) and enriches the research on negative workplace behaviors and wellbeing (73).

### 6.2. Practical implications

Since workplace ostracism affects employee wellbeing, managers should minimize or avoid ostracism in the workplace. For example, companies can reduce the incidence of ostracism by encouraging employees to use face-to-face communication, strengthening the care for employees through multiple ways, encouraging employees to participate in social activities, increasing communication opportunities between colleagues, improving emotional communication, promoting mutual understanding, and reduce the possibility of ostracism.

In addition, workplace ostracism consumes employees' emotional resources and causes emotional exhaustion. The organization should provide employees with more organizational support, such as a good communication environment and a cordial organizational atmosphere, which makes employees develop positive emotions toward work and helps them regain resources to overcome the negative effects of ostracism. At the same time, the organization can establish a psychological counseling mechanism for employees to reduce the level of emotional exhaustion and provide opportunities to replenish resources in an attempt to eliminate negative emotions.

Organizational culture has an inhibiting effect on team atmosphere. Therefore, the organization should create a healthy culture, construct a team climate of fairness, forgiveness, tolerance, and understanding, and develop an atmosphere of openness and trust among colleagues or between superiors and subordinates, to curb the occurrence of workplace ostracism at the source (74, 75). Certainly, if workplace ostracism has already occurred in an organization, this climate of forgiveness and trust can serve as a complementary resource to reduce the harmful effects of workplace ostracism.

## 7. Limitations and future research directions

The limitations of this study are as follows:

First, the paired data from leaders and employees were adopted in this study which can effectively reduce the influence of common method bias and make the results more credible. However, these are self-assessed questionnaires, and it is suggested that mutual valuation will be used to obtain survey data subsequently.

Second, the sample data of this study are all obtained from developed regions in mainland China, and whether the conclusions of this study are applicable to other regions and industries still needs further research.

Third, most of the scales used in this study were developed from the Western organizational background and have good reliability and validity. However, China believes in Confucianism and Zhong Yong Thinking (76). Therefore, it is suggested that future researchers can develop scales according to Chinese cultural background.

Fourth, this study uses organizational climate as a situational factor, but employee wellbeing is also influenced by personal traits and leadership (77–79). Therefore, it is suggested that future research could try to use personal traits and leadership as situational factors to more clearly reveal the impact of workplace ostracism on employee wellbeing.

## Data availability statement

The raw data supporting the conclusions of this article will be made available by the authors, without undue reservation.

## Author contributions

All authors listed have made a substantial, direct, and intellectual contribution to the work and approved it for publication.
